# Evaluation of calcium alginate hydrogel for suprachoroidal buckling in a rabbit model—a pilot study on efficacy and biocompatibility

**DOI:** 10.3389/fmed.2025.1632889

**Published:** 2025-07-25

**Authors:** Xingyu Xiao, Yan Zhou, Shiqun Lin, Huan Chen, Rongping Dai

**Affiliations:** ^1^Department of Ophthalmology, Peking Union Medical College Hospital, Chinese Academy of Medical Sciences, Beijing, China; ^2^Key Laboratory of Ocular Fundus Diseases, Chinese Academy of Medical Sciences & Peking Union Medical College, Beijing, China; ^3^Beijing Key Laboratory of Fundus Diseases Intelligent Diagnosis & Drug/Device Development and Translation, Beijing, China

**Keywords:** suprachoroidal buckling, calcium alginate hydrogel, rhegmatogenous retinal detachment, suprachoroidal space, ophthalmic surgery

## Abstract

**Purpose:**

This study aimed to evaluate the therapeutic efficacy, safety, and biocompatibility of calcium alginate hydrogel in suprachoroidal buckling procedures performed on rabbit eyes.

**Methods:**

A total of 21 healthy New Zealand White rabbits underwent surgery, during which calcium alginate hydrogel was injected into the suprachoroidal space of one eye. Monthly ophthalmic examinations were performed for 3 months following the procedure. The assessments included external eye and fundus examinations, color fundus imaging, and optical coherence tomography. At the end of the observation period, both eyes from each rabbit were enucleated and subjected to histopathological evaluation.

**Results:**

The surgery was successfully performed on 21 eyes, with a success rate of 80.95% (17/21). A total of 17 eyes showed favorable postoperative outcomes and no complications. The average maximum dome height is 2,470.42 ± 876.11 μm on the day of operation and 1,585.97 ± 351.93 μm 3 months postoperatively, with the average difference of 866.37 ± 592.94 μm. A total of four eyes experienced inadvertent perforations of both the choroid and retina, leading to vitreous and suprachoroidal hemorrhages. Histological evaluation confirmed the persistent presence of a suprachoroidal indentation in the treated eyes of the 17 rabbits without complications, with no observable abnormalities detected in the lenses or retinas.

**Conclusion:**

Calcium alginate hydrogel proved to be a safe and effective material for suprachoroidal buckling in rabbits, exhibiting excellent biocompatibility.

## Introduction

High myopia is a slow-progressing eye disease (1), which is a leading cause of visual impairment globally, with a remarkably rapid increase in prevalence across East Asia (2, 3). Among its vision-threatening complications are rhegmatogenous retinal detachment (RRD) and myopic traction maculopathy (MTM), both of which often necessitate surgical intervention. Common surgical treatments for RRD include pneumatic retinopexy (PnR), pars plana vitrectomy (PPV), and scleral buckling (SB), with PPV becoming the dominant approach over the past two decades. Nevertheless, SB has demonstrated superior functional outcomes in some cases (4, 5). Macular buckling (MB), on the other hand, has shown considerable promise in the management of MTM (6). However, traditional SB and MB techniques require placement of external implants—such as silicone bands or sponges—on the scleral surface to generate indentation. These materials can lead to postoperative complications, including restricted ocular motility and diplopia (7, 8). Suprachoroidal buckling (SCB) is an emerging alternative that mimics the indentation effect of SB/MB but utilizes the suprachoroidal space (SCS), which is a potential anatomical space located between the sclera and the choroid, as the site for device placement. By delivering filler materials directly into the SCS, SCB reduces vitreoretinal traction and prevents the ingress of fluid into the subretinal space in RRD, while also counteracting the posterior staphyloma-induced traction observed in MTM.

The concept of SCB dates back to 1986, when Poole and Sudarsky (9) first introduced the idea of injecting sodium hyaluronate into the SCS to achieve temporary apposition of the retina and choroid for RRD repair. However, this technique did not gain traction at the time. Over the ensuing decades, advances in ocular imaging and surgical instrumentation have reignited interest in SCB. More recent studies have demonstrated favorable outcomes, including high rates of retinal reattachment (10–13). One such innovation involved the use of a custom 30-gauge needle guard to enable in-office SCB, referred to as suprachoroidal viscopexy, for treating acute RRD (14), allowing the procedure to be performed in a minimally invasive manner. SCB has also been explored for MTM, where it has demonstrated therapeutic efficacy (15). While there are clear advantages to this procedure, including its minimally invasive nature, the use of absorbable filling materials, and the potential to avoid complications such as episcleral buckles (11–13), SCB has yet to be widely adopted, primarily due to unresolved concerns regarding safety, a steep learning curve, and limited clinical experience (16), emphasizing a need for further study.

Currently, hydrogels are the predominant filler materials used in SCB. Healon5, a commonly utilized option, maintains its buckling effect for approximately 2.6 weeks (17). However, cases involving substantial serum response factor (SRF) or chronic MTM may require more prolonged or even permanent indentation. Alginate hydrogel, an inert biomaterial, has shown long-term stability in prior studies, including implantation into the left ventricular myocardium in heart failure patients (18). This has led us to investigate its potential as a long-lasting, biocompatible filler for SCB. Notably, existing studies have not sufficiently addressed the long-term safety, efficacy, and tissue compatibility of alginate hydrogel in ophthalmic applications. To address this gap, the present study was developed to evaluate the therapeutic efficacy, safety profile, and biocompatibility of calcium alginate hydrogel when used for SCB in a rabbit model. This evaluation includes ophthalmic imaging, histological assessments, documentation of complications and their natural resolution, and surgical technique refinements to optimize outcomes.

## Methods

### Materials

Calcium alginate hydrogel was sourced from Deke Medicine (Hangzhou, China). Based on prior rheological analysis, the hydrogel exhibited a loss factor of 0.55 and a complex viscosity of 0.34931. A total of 21 healthy New Zealand White rabbits, each aged 8 weeks and weighing between 1.0 and 1.5 kg, were included in the study. Surgical intervention was performed on one eye per rabbit (randomly assigned to either the right or left side) by two experienced retinal surgeons (HC and RPD), with the contralateral eye serving as the untreated control. Animal care protocols complied with the Association for Research in Vision and Ophthalmology (ARVO) Statement for the Use of Animals in Ophthalmic and Vision Research. They were approved by the Experimental Animal Welfare Ethics Committee of Peking Union Medical College Hospital. Under general anesthesia, induced by a single intramuscular injection of 0.1 mL/kg, calcium alginate hydrogel was introduced into the suprachoroidal space to form a localized buckle in the superior nasal quadrant.

### Surgical procedure

**Figure 1:**
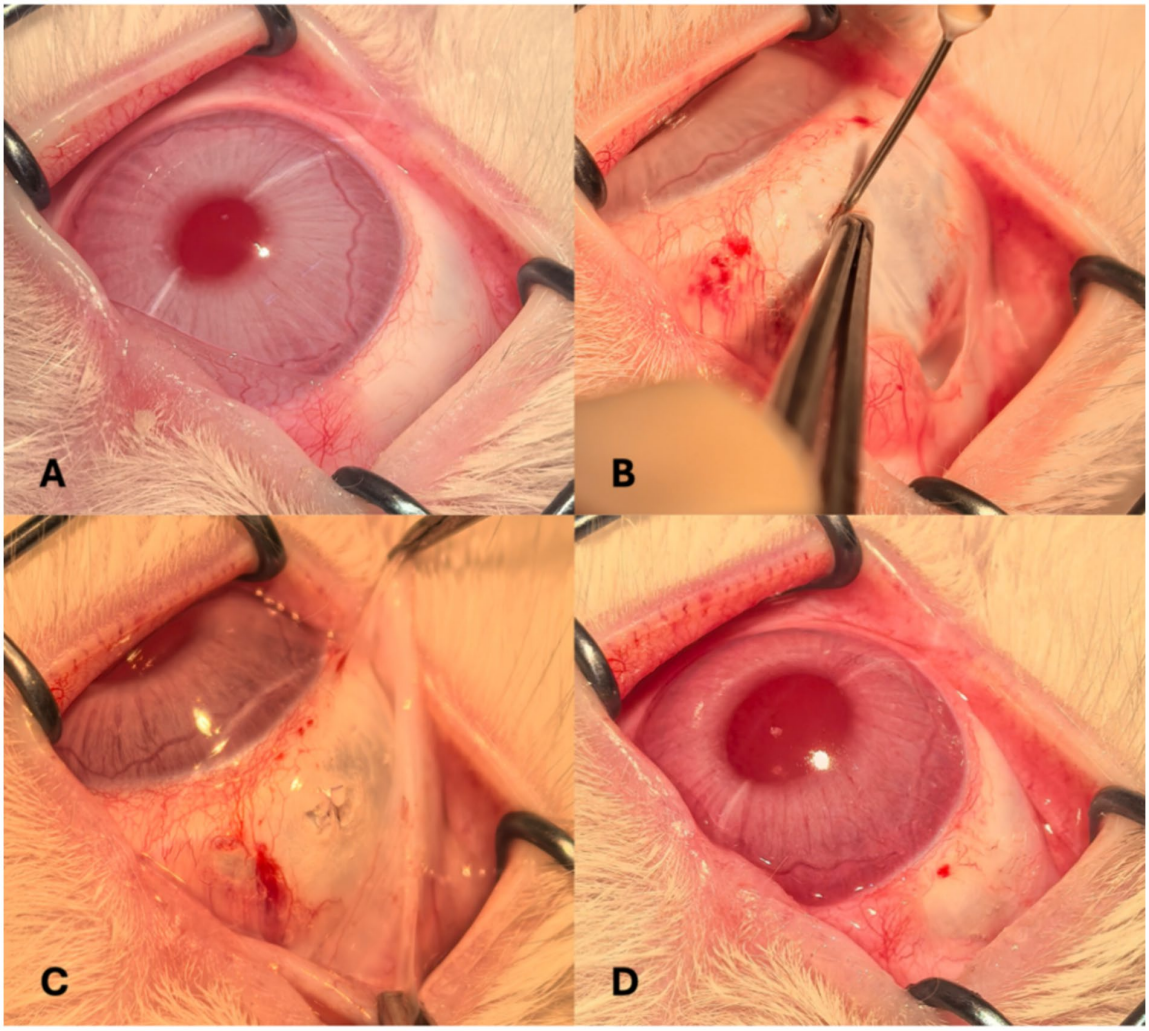
External ocular photographs taken before **(A)**; during **(B)** and **(C)**; and after the procedure **(D)**.

The operative site was prepared by sterilizing the conjunctival sac with a povidone-iodine solution for 30 s, followed by a rinse with sterile saline. A conjunctival incision was made in the superior nasal quadrant, and a 3-mm circumferential sclerotomy was created approximately 3 mm posterior to the limbus to expose the underlying choroid. A 7–0 Vicryl suture (Ethicon, Somerville, NJ, USA) was preplaced at the wound site. Following choroidal exposure, a viscoelastic agent (IVIZ; 1% sodium hyaluronate, Shandong Zhengdafeida Pharmaceutical Co., Ltd., Shandong, China) was injected at the posterior margin of the sclerotomy to form a suprachoroidal pocket. To manage intraocular pressure (IOP), anterior chamber paracentesis was performed simultaneously. A catheter was then inserted through the sclerotomy and advanced approximately 3 cm posteriorly within the SCS. Once properly positioned, 0.1–0.2 mL of calcium alginate hydrogel was slowly injected. Upon completion of the injection, the catheter was carefully withdrawn, and the sclerotomy was closed by tightening the preplaced suture. IOP was reassessed at the end of the procedure. Tobramycin-dexamethasone ointment was applied to the conjunctival sac to minimize inflammation and infection risk. Representative pre-, intra-, and postoperative external eye images are presented in Figure 1.

### Postoperative examination

Comprehensive ocular evaluations were performed monthly for a duration of 3 months following surgery. These assessments included external ocular inspection, fundus examination, and IOP measurement. External evaluations focused on signs of postoperative inflammation and wound healing. Optical coherence tomography (OCT) and color fundus imaging were employed to assess the height of the dome-shaped buckle and to monitor retinal condition. OCT scans were obtained using a VG200 swept-source OCT system (SVision Imaging, Ltd., Luoyang, China), while fundus images were captured with a confocal scanning laser ophthalmoscope Optos® 200Tx (Optos Plc, Scotland, United Kingdom). For each imaging session, rabbits were anesthetized, and mydriasis was induced with 1% tropicamide ophthalmic drops. At the conclusion of the 3-month observation period, both eyes from each rabbit were enucleated for histopathological evaluation, including hematoxylin and eosin (H&E) staining, to determine the biocompatibility of the implanted material.

## Results

### Gross observations

The surgical procedure was successfully conducted in 21 eyes from 21 rabbits. The primary outcome indicators are success buckling rate, average maximum dome height on the day of operation, and 3 months postoperatively. The success buckling is defined as the obvious chorioretinal indentation with an initial maximum dome height greater than twice the chorioretinal thickness, and the indentation persists for more than 3 months. We have calculated the success buckling rate of 80.95% (17/21) and the average maximum dome height of 2470.42 ± 876.11 μm on the day of operation and 1585.97 ± 351.93 μm 3 months postoperatively, with the average difference of 866.37 ± 592.94 μm. Throughout the postoperative observation period, there were no signs of anterior chamber inflammation or hydrogel leakage in any of the subjects. Intraocular pressure (IOP) exhibited a transient elevation immediately following surgery but returned to baseline values by the following day and remained within the normal range thereafter. Conjunctival hyperemia gradually subsided over time. Of the 21 cases, 17 eyes underwent the procedure without complications and demonstrated favorable postoperative outcomes. However, four eyes experienced inadvertent perforation of both the choroid and retina, resulting in vitreous and suprachoroidal hemorrhages.

### Fundus examination

**Figure 2:**
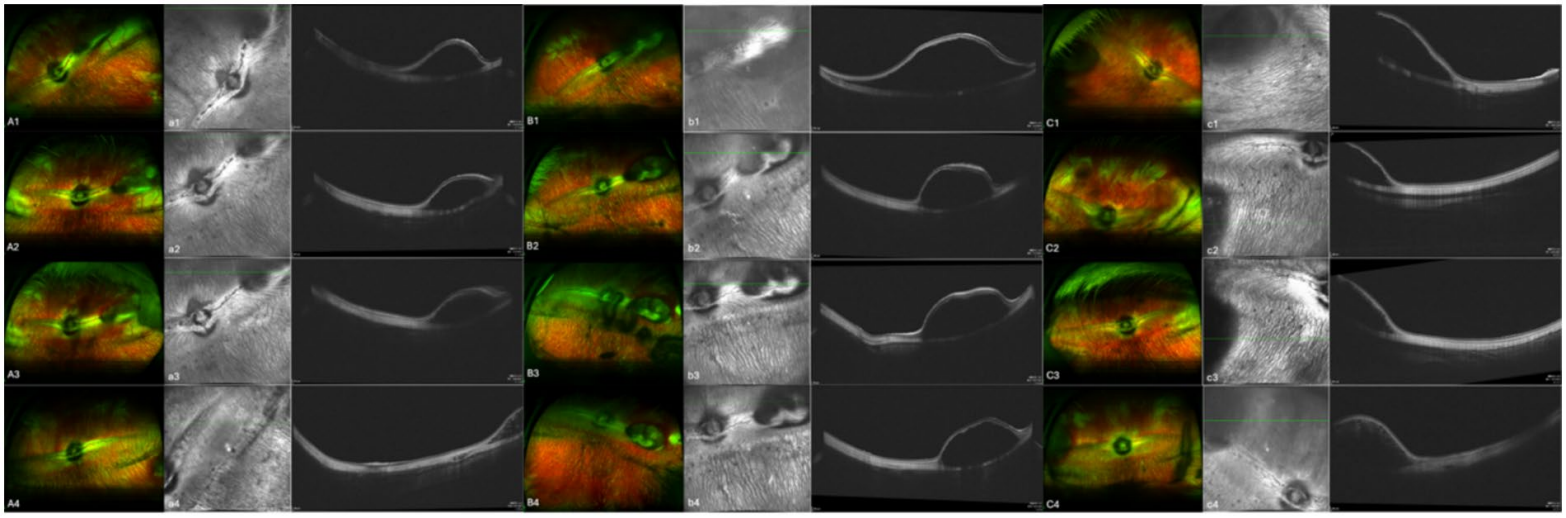
Aa, Bb, and Cc show images from three separate eyes. A, B, and C are fundus color images; a, b, and c are the corresponding OCT images. Images 1, 2, 3, and 4 were taken on the day of surgery, at 1 month post-surgery, at 2 months post-surgery, and at 3 months post-surgery, respectively.

**Figure 3:**
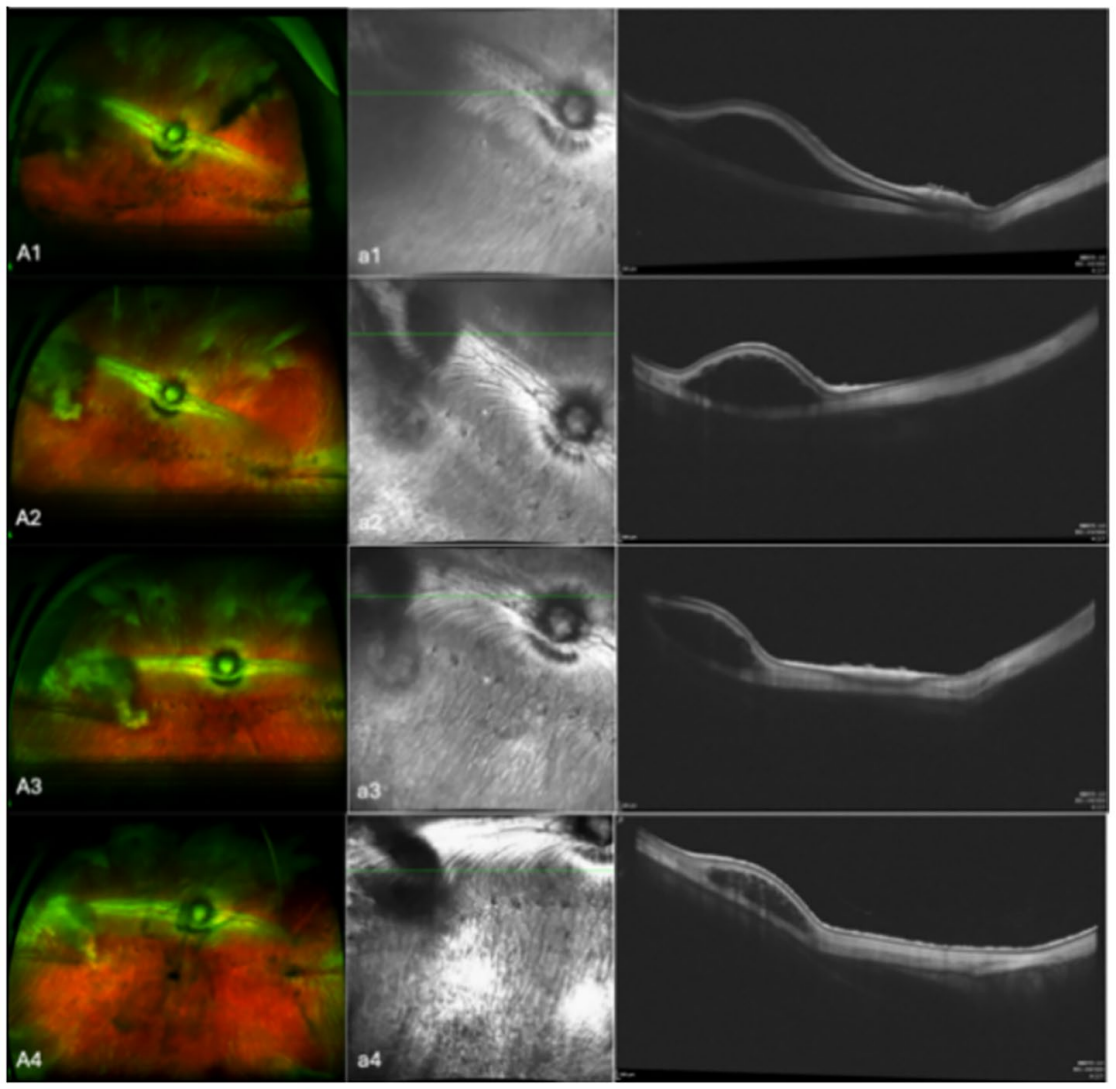
Aa shows representative images from eyes that OCT revealed high-reflectivity signals in the suprachoroidal space. A are fundus color images; a are the corresponding OCT images. Images 1, 2, 3, and 4 were taken on the day of surgery, at 1 month post-surgery, at 2 months post-surgery, and at 3 months post-surgery, respectively.

**Figure 4:**
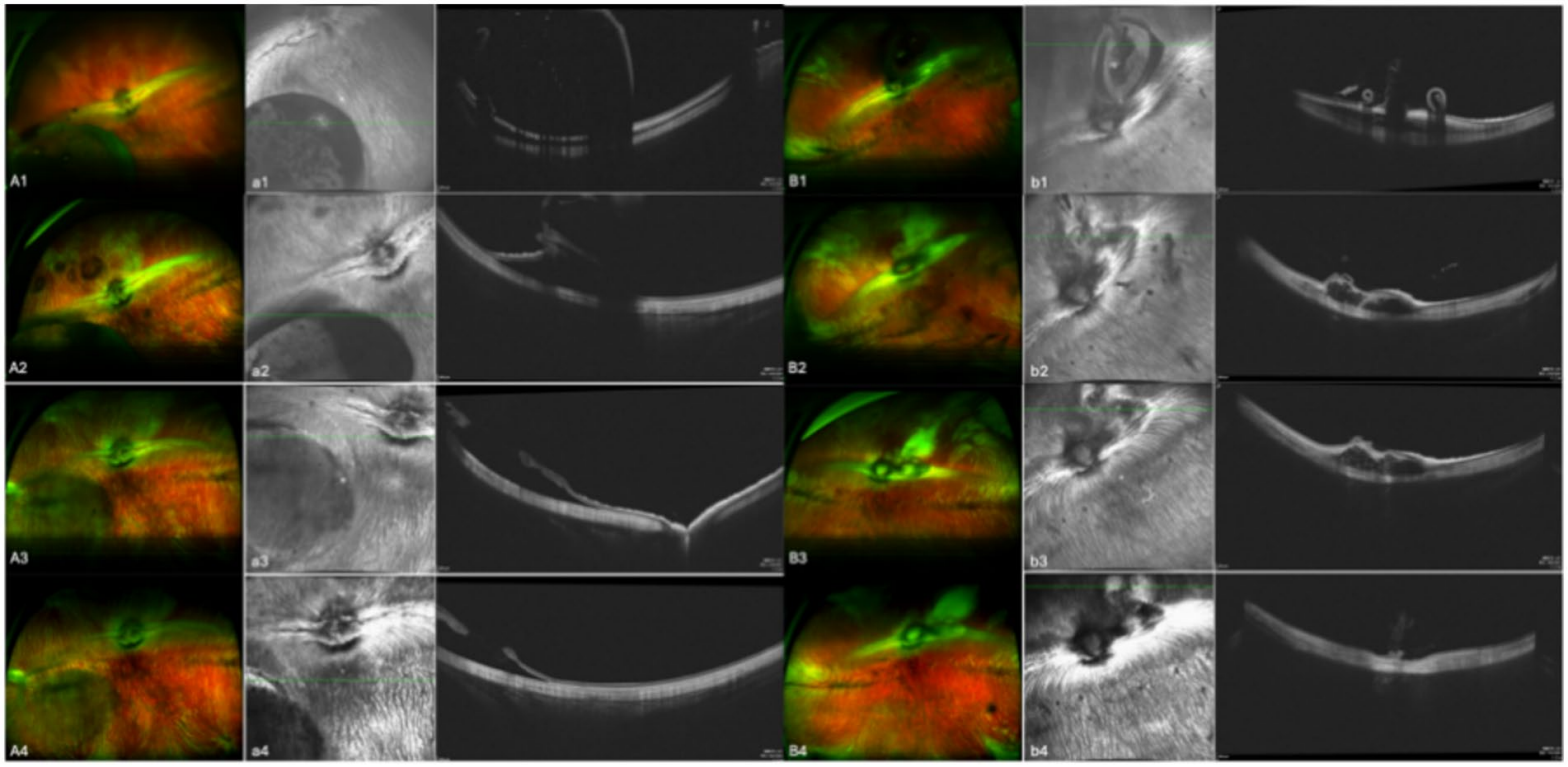
Aa and Bb show images from two separate eyes. A and B are fundus color images; a and b are the corresponding OCT images. Images 1, 2, 3, and 4 were taken on the day of surgery, at 1 month post-surgery, at 2 months post-surgery, and at 3 months post-surgery, respectively. Bb shows the eye in which choroidal retinal perforation closed over the course of follow-up, whereas Aa shows the eye in which this did not occur.

In the 17 eyes where the suprachoroidal hydrogel buckling procedure was deemed successful, localized choroidal indentation persisted for over 3 months postoperatively. The depth of the indentation was primarily determined by the volume of hydrogel injected. These indentations remained stable and smooth during the entire 3-month observation period, with no significant signs of hydrogel resorption or migration. Fundus photography did not reveal any complications such as retinal or choroidal hemorrhages or detachments. Representative fundus images from three of the 17 successfully treated eyes are presented in Figure 2. During follow-up, OCT revealed persistent high-reflectivity signals in the suprachoroidal space in several eyes, with these signals being most prominent at the 3-month mark (Figure 3). Among the four eyes that sustained chorioretinal perforations, suprachoroidal and vitreous hemorrhages were observed. Over the course of follow-up, two of these cases exhibited spontaneous closure of the perforation sites, accompanied by reattachment of the choroid and retina. Representative images from two affected eyes are shown in Figure 4.

### Histologic analysis

**Figure 5:**
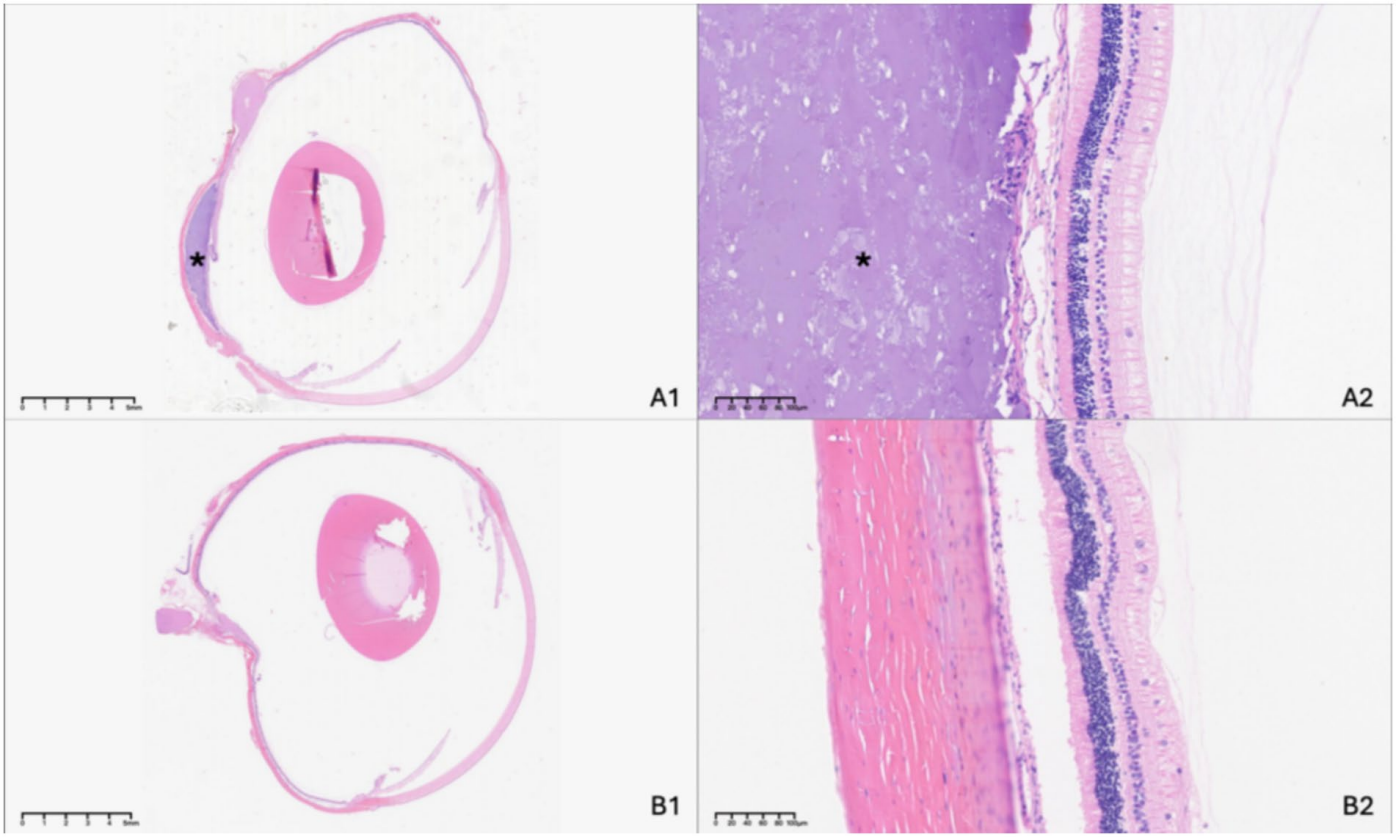
Histologic examination of the experimental eye at 3 months following suprachoroidal hydrogel buckling **(A)**, with the contralateral eye serving as the normal control **(B)**. The asterisk (*) indicates the site of the hydrogel buckle.

Histological evaluation conducted 3 months post-surgery confirmed the sustained presence of a suprachoroidal indentation in treated eyes of the 17 rabbits without complications, with the lenses remaining transparent, and retinal architecture appeared intact with no observable histopathological abnormalities. Neither significant inflammatory cell infiltration nor fibrotic response to the implant could be observed compared to the unoperated control eye (Figure 5).

## Discussion

The optimal approach to managing RRD continues to be the subject of considerable debate and evolving clinical practice patterns (19). Despite comparable anatomic and visual outcomes between SB and PPV (20), SB has been associated with a lower incidence of postoperative retinal displacement (21). Moreover, the rapid drainage of SRF and the forceful juxtaposition of the retina with the rate of perceived exertion (RPE) during PPV may impede the optimal restoration of the photoreceptor-RPE interface. SB, in contrast, permits gradual SRF reabsorption via the RPE, potentially leading to superior retinal reattachment and functional recovery (4). At present, silicone-based permanent implants are widely utilized in SB procedures due to their chemical inertness, biocompatibility, and structural stability (22). However, these materials are not without limitations. Drawbacks include prolonged healing time, ocular discomfort, and the risk of long-term complications such as induced refractive errors, ocular motility disorders (e.g., strabismus and diplopia), anterior segment ischemia, and rare but serious complications like infection and conjunctival extrusion (7, 23, 24). Additionally, the technical demands and time-intensive nature of SB have contributed to a decline in its use, especially among younger ophthalmic surgeons who often lack extensive training in this technique (20, 25). MB has demonstrated efficacy in the treatment of MTM but is also accompanied by a range of complications (6, 8). While the development of absorbable buckling materials is underway, SCB introduces a novel therapeutic strategy for both RRD and MTM. This method involves injecting a filler into the potential space between the sclera and choroid to achieve a buckling effect, thereby leveraging a natural anatomical compartment.

Since Poole’s initial application of SCB for RRD, numerous studies have investigated its safety and efficacy in selected patient populations (10, 12, 13, 17). Early SCB procedures employed various filler materials, such as air, gelatin sponges, plasma, and coagulated fibrin, but these substances were eventually abandoned due to their limited practicality and suboptimal outcomes (9). More recent studies have favored hydrogel materials, particularly Healon5, as SCB fillers. These hydrogels have demonstrated reliable retention within the suprachoroidal space for an average duration of 2.6 weeks (12, 17). Notably, persistent SRF has been observed in 55% of RRD patients even 6 weeks after SB surgery (26). While there is no standardized optimal duration for indentation, brief buckling periods may result in inadequate chorioretinal adhesion due to persistent SRF and incomplete laser scar formation. Therefore, achieving a long-lasting indentation is critical for successful retinal reattachment, especially in cases involving extensive or high-grade detachments, which necessitate the use of high-strength, slow-resorbing hydrogels. In the context of MTM, a durable macular buckle may mitigate axial elongation and limit the progressive outward expansion of the retina, RPE, and choroid, thereby slowing the progression of myopic degeneration (8, 27). Among candidate materials, alginate-based hydrogels offer tunable mechanical and biochemical properties and have been widely employed in biomedical applications due to their excellent biocompatibility (28). Increasing the degree of cross-linking between calcium ions (Ca^2+^) and sodium alginate enhances hydrogel stability, making these formulations more resistant to deformation and degradation (29). As a result, calcium alginate hydrogels have been extensively utilized as bulking agents, as sealants, as promoters of wound healing, and in angiogenesis applications (30). A novel calcium alginate hydrogel developed by Deke Medicine (Hangzhou, China) has been previously evaluated in a clinical study involving direct myocardial implantation in patients with heart failure, where it demonstrated both safety and therapeutic efficacy (18). Rheological testing of this hydrogel showed low flowability and strong structural integrity, properties that are desirable for maintaining a consistent shape and position *in vivo*. In the present study, we explored the potential application of hydrogel in SCB by injecting it into the suprachoroidal space to create a long-lasting indentation. To our knowledge, this represents the first use of calcium alginate hydrogel in an SCB procedure. Nevertheless, the long-term safety of sustained suprachoroidal hydrogel compression remains to be fully elucidated. In particular, more extensive animal studies are needed to confirm the biocompatibility and long-term tolerability of this material when used in the ocular environment.

In this study, we developed a minimally invasive SCB technique using a novel calcium alginate hydrogel in a rabbit model, achieving promising surgical outcomes. Successful SCB procedures were performed in 17 out of 21 eyes, all of which demonstrated favorable postoperative results without any observed complications during the follow-up period. Notably, a sustained, localized choroidal indentation was evident for over 3 months post-surgery. Fundus photography throughout the follow-up period revealed no signs of ischemia. This absence of ischemic damage is likely attributable to the hydrogel’s localized indentation effect, which applies minimal mechanical stress to the choroidal vasculature, thereby preserving choroidal circulation. Although indocyanine green angiography (ICGA) would provide more definitive insights into vortex vein perfusion, no choroidal thickening or subretinal fluid, commonly associated with excessive choroidal compression (8, 31, 32), was observed in the operated eyes when compared to the contralateral controls. These results suggest that SCB may avoid or significantly reduce complications related to the direct compression caused by episcleral implants, especially those with sharp edges. Histological examination further confirmed the biocompatibility of the hydrogel. Among the 17 eyes, the lenses remained clear, retinal architecture was preserved without structural disruption, and neither significant inflammatory cell infiltration nor fibrotic response to the implant could be observed. Additionally, the histological evidence indicated a persistent buckling effect lasting at least 3 months postoperatively. Interestingly, in several cases, OCT imaging revealed heterogeneous hyperreflective signals in the suprachoroidal space, especially at the 3-month mark. These findings, supported by histological analysis, suggest the formation of small hydrogel particles or irregular interface regions within the material, potentially due to variations in refractive index relative to surrounding tissues. Further investigations using confocal or electron microscopy may elucidate the microstructural characteristics of the hydrogel. Previous studies have reported elevated IOP following the SB and MB procedures (8, 33). To mitigate such risks, we performed anterior chamber paracentesis prior to hydrogel injection to facilitate smoother delivery and achieve optimal choroidal indentation. No postoperative paracentesis was required. Although a transient increase in IOP was observed immediately after surgery, it normalized by the following day and remained within physiological limits thereafter. These findings suggest that SCB exerts minimal influence on aqueous humor dynamics and IOP regulation. Furthermore, SCB was found to be less time-intensive than traditional episcleral buckling. Beginning with the fourth case, the average time from conjunctival incision to scleral suturing was reduced to under 10 min. However, this time measurement did not include the localization of retinal breaks or verification of retinal reattachment. Several labor-intensive steps common to SB, such as extensive conjunctival peritomy, extraocular muscle manipulation, buckle placement and fixation, and conjunctival closure, were omitted, rendering SCB a more streamlined and less invasive approach.

Regarding complications, four eyes experienced accidental choroidal-retinal perforation, resulting in vitreous and suprachoroidal hemorrhage in the present study. It is remarkable that, in two of these cases, spontaneous closure of the perforation was observed during follow-up, with subsequent reattachment of both the retina and choroid. We postulate that the hydrogel’s viscous properties contributed to sealing the choroidal-retinal breaks and promoting tissue reapposition. Similar effects have been noted with ophthalmic viscosurgical devices (OVDs), which have been safely used to stabilize internal limiting membrane flaps during macular hole surgery (34). Additionally, recent innovations such as PYK-2101, a patented biodegradable hydrogel designed to seal retinal breaks without requiring intraocular gas or oil, underscore the potential of viscous materials in retinal repair. It is important to consider anatomical differences, as the rabbit sclera and choroid are thinner than those of humans, which may influence outcomes. The current injection apparatus used to deliver the hydrogel and induce buckling is relatively invasive. However, the future adoption of non-invasive catheter systems is expected to enhance procedural safety (12, 16). Despite these advancements, risks such as those associated with high myopia, as well as the steep learning curve of SCB, must not be overlooked. In prior clinical studies, hemorrhagic complications, most notably suprachoroidal hemorrhage and subretinal hemorrhage, were among the most common adverse events. For example, Antaki et al. (16) reported a hemorrhage incidence of 23% (six out of 26 cases). In our study, minor choroidal hemorrhage was noted in three eyes during scleral incision to expose the choroid. These hemorrhages resolved with direct pressure and did not disrupt the procedure. All instances occurred early in the study, likely due to the inadequate control of incision depth. No fundus hemorrhages were detected in any of the 17 eyes where successful buckling was achieved.

Among the key strengths of this study are the demonstration of prolonged indentation using calcium alginate hydrogel, direct visualization and assessment of SCB morphology through OCT imaging rather than traditional B-scan ultrasonography or CT, and the use of histological analysis to evaluate biocompatibility. However, several limitations should be acknowledged. First, we used healthy rabbits rather than models with retinal detachment, thus efficacy claims relate only to mechanical buckling, but not to disease resolution, although many animal studies on SB have similarly used healthy subjects (35). Second, the differences in the scleral and choroidal circulation between rabbits and human also limits the applicability of the research and thus warrants further study. Besides, this was a pilot study with a limited sample size, lacking rigorous experimental controls and more accurate quantitative indicators. Nevertheless, the insights gained in the study can serve as a foundation for future research. Finally, the injection device used in this study is relatively invasive, though alternative non-invasive delivery systems are under development.

## Conclusion

Our findings demonstrate that calcium alginate hydrogel-based SCB offers promising efficacy and biocompatibility in a rabbit model, with choroidal indentation persisting for over 3 months. These results suggest that this technique holds potential as a future alternative or adjunctive treatment for rhegmatogenous retinal detachment or myopic traction maculopathy. However, additional studies are necessary to confirm its effectiveness in pathological conditions. Given the potential for complications and the procedural learning curve, cautious patient selection and surgeon training remain crucial. The adoption of advanced visualization tools and minimally invasive delivery devices will likely enhance both the safety and feasibility of SCB in clinical practice.

## Data Availability

The original contributions presented in the study are included in the article/supplementary material, further inquiries can be directed to the corresponding authors.
